# Database Mining of Genes of Prognostic Value for the Prostate Adenocarcinoma Microenvironment Using the Cancer Gene Atlas

**DOI:** 10.1155/2020/5019793

**Published:** 2020-05-18

**Authors:** Xin Zhao, Daixing Hu, Jia Li, Guozhi Zhao, Wei Tang, Honglin Cheng

**Affiliations:** ^1^Department of Urology, The First Affiliated Hospital of Chongqing Medical University, Chongqing Medical University, Chongqing, China; ^2^Department of Urology, The Affiliated Hospital of Southwest Medical University, Luzhou, China; ^3^Department of Thyroid and Breast, Shanghai Tenth People's Hospital, Tongji University, School of Medicine, Shanghai 200072, China

## Abstract

**Background:**

Prostate adenocarcinoma (PRAD) is a common malignant tumor in elderly men. Our research uses The Cancer Gene Atlas (TCGA) database to find potential related genes for predicting the prognosis of patients with PRAD.

**Methods:**

We downloaded gene expression profiles and clinical sample information from TCGA for 490 patients with PRAD (patient age: 41-78 years). We calculated stromal and immune scores using the ESTIMATE algorithm to predict the level of stromal and immune cell infiltration. We categorized patients with PRAD in TCGA into high and low score arrays according to their median immune/stromal scores and identified differentially expressed genes (DEGs) that were significantly correlated with the prognosis of PRAD. Then, Gene Ontology (GO) and Kyoto Encyclopedia of Genes and Genomes (KEGG) pathway analyses were performed. The association between DEGs and overall survival was investigated by weighted Kaplan–Meier survival analysis and multivariate analysis. Furthermore, the protein-protein interaction network (PPI) of DEGs was constructed using the STRING tool. Finally, the hub genes were identified by analyzing the degree of association of PPI networks.

**Results:**

We found that 8 individual DEGs, C6, S100A12, MLC1, PAX5, C7, FAM162B, CAMK1G, and TCEAL5, were significantly predictive of favorable overall survival and one DEG, EPYC, was associated with poor overall survival. GO and KEGG pathway analyses revealed that the DEGs were associated with immune responses. Moreover, 30 hub genes were obtained using the PPI network of DEGs: ITGAM, CD4, CD3E, IL-10, LCP2, ITGB2, ZAP-70, C3, CCL19, CXCL13, CXCL9, BTK, CCL21, CD247, CD28, CD3D, FCER1G, PTPRC, TYROBP, CCR5, ITK, CCL13, CCR1, CCR2, CD79B, CYBB, IL2RG, JAK3, PLCG2, and CD19. These prominent nodes had the most associations with other genes, indicating that they might play crucial roles in the prognosis of PRAD.

**Conclusions:**

We extracted a list of genes associated with the prostate adenocarcinoma microenvironment, which might contribute to the prediction and interpretation of PRAD prognosis.

## 1. Introduction

Prostate cancer is the second leading cause of cancer deaths in American men [1]. With increasing living standards in China, the incidence of prostate cancer has also increased over time [2]. Prostate adenocarcinoma (PRAD) is the most common type of prostate cancer, whereas other types of prostate cancer are relatively rare [3, 4]. At present, the initial screening of subjects with suspected prostate cancer mainly involves the detection of serum prostate-specific antigens [5]. The current treatment methods for prostate cancer include surgery, radiotherapy, chemotherapy, and endocrine therapy, but the prognosis differs among various patients [6, 7]. Some patients with prostate cancer can survive for 10–20 years after treatment, whereas others respond poorly to treatment and die because of metastasis within 2–3 years. Currently, only few methods are available for evaluating and predicting the prognosis of PRAD. Therefore, there is an urgent need to determine a rapid and noninvasive method for predicting the prognosis of diseases in such patients. The establishment of TCGA has provided us with a wealth of clinical data and has enabled us to discover genomic abnormalities in several populations worldwide [8, 9]. Moreover, we can further understand the influence of human genome on clinical prognosis.

Tumors have an extremely complex microenvironment comprising stromal cells, immune cells, inflammatory mediators, and extracellular matrix (ECM) [10, 11]. Previous studies have indicated that the tumor microenvironment plays a crucial role in influencing gene expression in tumor tissues [12]. In the tumor microenvironment, stromal and immune cells represent two primary nontumor components, which are important for the diagnosis and prognosis of tumors [13]. Yoshihara et al. [14] have described “Estimation of STromal and Immune cells in MAlignant Tumor tissues using Expression data” (ESTIMATE), a method for inferring the proportions of stromal and immune cells in a tumor sample using gene expression characteristics. Furthermore, the functional enrichment of single sample genes can be predicted using GO and KEGG pathway analyses [15, 16]. Yoshihara et al. [14] have predicted the levels of infiltrating stromal and immune cells by calculating immune and stromal scores, which formed the basis for calculating the ESTIMATE score of tumor purity for tumor tissues. Subsequent reports applied this estimation method to breast [17] and rectal cancers [18] and demonstrated the effectiveness of this algorithm.

In this study, for the first time, we used TCGA data of patients with PRAD to identify a set of tumor microenvironment-related genes, which are predictive of poor prognosis in these patients. The results revealed a list of genes, thus providing a better understanding of this disease and further clarifying the relationship between prognosis and the tumor microenvironment in patients with PRAD.

## 2. Methods and Materials

### 2.1. Database

The clinical information of patients with PRAD was downloaded from TCGA (https://tcga-data.nci.nih.gov/tcga/), and their clinicopathological information was extracted from the atlas data portal. The ESTIMATE algorithm was applied to the downloaded data to calculate the immune and stromal scores [14].

### 2.2. Identification of DEGs

The PRAD patients were split into high and low immune/stromal score groups based on the median immune/stromal scores. DEGs were analyzed using the limma package between the high and low immune/stromal score groups. The cutoff points for screening DEGs were fold changes of >1.5 and adjusted *p* < 0.05 [19].

### 2.3. Integration of PPI Networks

The Search Tool for the Retrieval of Interacting Genes/Proteins (STRING) database (http://www.strindb.org/) is a well-known network of tools for examining PPIs [20]. All the 524 intersected genes between the immune and stromal score groups were mapped into STRING to assess the relationship among these genes.

### 2.4. GO and Functional Analyses

GO and KEGG pathway enrichment analyses were performed using the R Project for Statistical Computing (version 3.6.1; https://www.r-project.org/) [21] to characterize the functional enrichment of all DEGs. GO describes the information based on knowledge of a biological domain in relation to three components: biological process (BP), molecular function (MF), and cellular component (CC). DEGs were considered to be significantly enriched in GO terms or KEGG pathways using the cutoffs of false discovery rate-adjusted *p* value < 0.05.

### 2.5. Statistical Analyses

Pearson correlation was used to analyze the association between T clinical stage and immune or stromal scores. Considering the confounders such as age and sample size might affect the correlation analysis, we used the linear regression model to analyze the association between immune scores and cancer stage as well as possible confounders age and sample size. Weighted Kaplan–Meier survival analysis was performed using the svykm function of R package of survey [22]. Difference in the surviving probabilities was compared with the function of svylogrank of R package of survey [22]. Multivariate analysis was conducted between overall survival, gene expression, or immune/stromal scores and patient's age using the svycoxph function of R package of survey [22]. Odds ratio and *p* values were extracted from the proportional hazards model. *p* value less than 0.05 was considered statistically significant.

## 3. Results

### 3.1. Immune and Stromal Scores Significantly Correlated with the T Clinical Stage of PRAD

**Figure 1:**
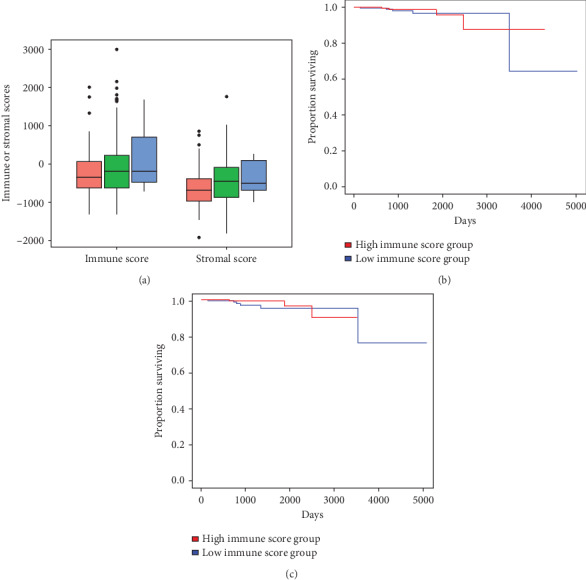
Immune and stromal scores are significantly correlated with the T clinical stage of PRAD patients. (a) Distribution of immune and stromal scores by the T clinical stage of PRAD. Boxplot showed a significant correlation between the T clinical stage of PRAD and the immune and stromal scores (*n* = 490). (b) Patients with PRAD were divided into two groups based on their median immune scores: high immune score group (*n* = 246), low immune score group (*n* = 244). Kaplan–Meier survival curves revealed that the median overall survival was higher in the low immune score group. (c) The median overall survival was higher in the group of patients with low stromal scores than in those with higher stromal scores, but no significant difference was observed between the two groups.

We downloaded gene expression profile and clinical sample information from TCGA for 490 patients with PRAD (patient age: 41–78 years). All the cases of PRAD with complete gene expression data and clinical information in TCGA were analyzed (supplementary Table 1). As shown in Figures 1(a) and 1(b), according to the ESTIMATE algorithm, immune scores ranged from −1344 to 3002.89 and stromal scores ranged from −1929.58 to 1762.30. The rank order of immune scores across T clinical stages was T4>T3>T2 (Figure 1(a), *p* < 0.05). Similarly, the median stromal scores were the highest for clinical stage T3 among all the clinical stages, followed by T4 (*p* < 0.01, Figure 1(b)). The T clinical stage was significantly positively correlated with stromal score (correlation coefficient: 0.18, *p* value < 0.001, Pearson correlation) and immune score (correlation coefficient: 0.14, *p* value < 0.01, Figure 1(a), Pearson correlation). Considering the confounders such as age and sample size might affect the correlation analysis, we used the linear regression model to analyze the association between immune scores and cancer stage as well as possible confounders age and sample size. As shown in the supplementary Table 2, the T clinical stage still exhibited significant association with immune scores with the addition of confounders (*p* < 0.05 for all cases).

To explore the potential correlations of overall survival with immune and/or stromal scores in patients with PRAD, the patients were divided into high and low immune score groups based on the median immune and/or stromal scores. Weighted Kaplan–Meier survival analysis (Figure 1(b)) revealed that the median overall survival was longer in the low immune score group (*p* = 0.49, weighted log-rank test) than that in the high immune score group. Consistently, the median overall survival was similarly prolonged in the low stromal score group (*p* = 0.28, weighted log-rank test, Figure 1(c)). However, the differences were not significant. Multivariate analysis results demonstrated that the higher immune and/or stromal scores were associated with an increase in mortality (*p* = 0.81, odds ratio (OR): 0.86, 95% Confidence Interval (CI): 0.24–3.08 for immune score, *p* = 0.28, OR: 0.46, 95% CI: 0.11–1.89 for stromal score).

### 3.2. Comparison of PRAD Gene Expression Profiles according to the Immune and Stromal Scores

**Figure 2:**
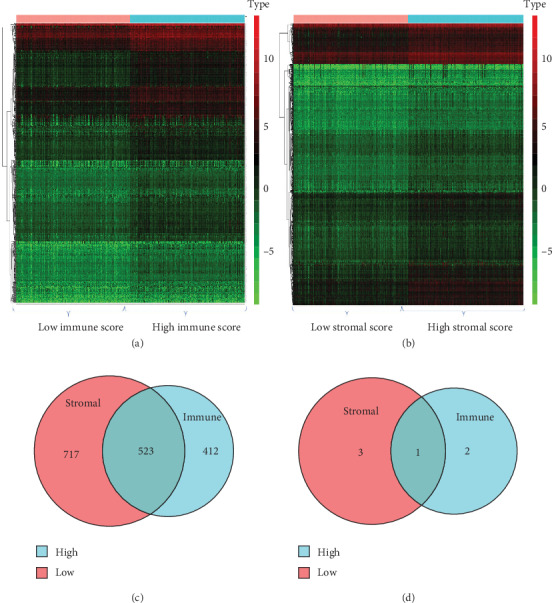
Comparison of prostate adenocarcinoma (PRAD) gene expression profile according to immune and stromal scores. The average linkage method and Pearson distance measurement method were used to draw the heat maps. The genes with high expression are shown in red, those with low expression are shown in green, and those with the same expression are shown in black. (a) A heat map of DEGs (fold change > 1.5, *p* < 0.05) between the high and low immune score groups. (b) Heat map of DEGs (fold change > 1.5, *p* < 0.05) between the high and low stromal score groups. (c, d) Venn diagrams showed the (c) upregulated genes for the high immune and high stromal groups and the overlap representing genes common to the high stromal group versus the high immune group (d) downregulated genes for the high immune and high stromal groups and the overlap representing genes common to the high stromal group versus the high immune group.

To determine whether global gene expression profiles are related to the immune and/or stromal scores, we compared the Affymetrix microarray data of 490 patients. The gene expression profiles of the high and low immune and stromal score groups are presented as heat maps in Figures 2(a) and 2(b). Regarding immune scores, 935 genes were upregulated in the high immune score group, whereas 3 genes were downregulated (fold change > 1.5, *p* < 0.05). Similarly, 1240 genes were upregulated in the low immune score group, whereas 4 genes were downregulated. In addition, as shown in Figures 2(c) and 2(d), Venn diagrams indicated that 523 upregulated genes were common to the high immune and stromal score groups; only 1 downregulated gene in the high immune score group was overlapped with that in the high stromal score group.

### 3.3. GO Term Analysis of DEGs Obtained from TCGA

**Figure 3:**
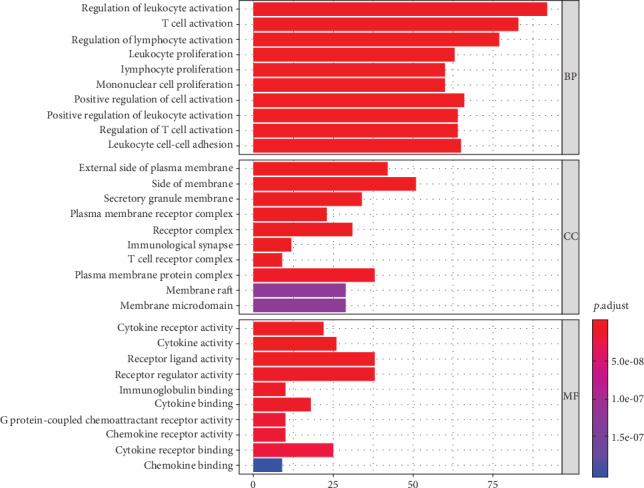
GO term enrichment analysis of DEGs obtained from TCGA. The main GO terms (false discovery rate-adjusted *p* values < 0.05) are shown for biological process, cellular component, and molecular function.

To explore the potential functions of DEGs, we analyzed the functional enrichment of GO terms for 935 upregulated genes in the high immune score group. Regarding BP, DEGs were enriched in the regulation of leukocyte activation, T-cell activation, lymphocyte activation, leukocyte proliferation, lymphocyte proliferation, and mononuclear cell, as well as in the proliferation positive regulation of cell activation, positive regulation of leukocyte activation, regulation of T-cell activation, and leukocyte cell–cell adhesion. For CC, DEGs were enriched in the external side of the plasma membrane, side of the membrane, secretory granule membrane, plasma membrane receptor complex, and receptor complex. For MF, DEGs were enriched in cytokine receptor activity, cytokine activity, receptor ligand activity, receptor regulator activity, immunoglobulin binding, and cytokine binding. Our results suggest that the functional clusters of genes exhibit strong correlations with immune responses (Figure 3). We also conducted the GO term enrichment analysis for the 3 downregulated genes; however, the genes were not significantly enriched in any GO terms.

### 3.4. KEGG Pathway Analysis of DEGs Obtained from TCGA

**Figure 4:**
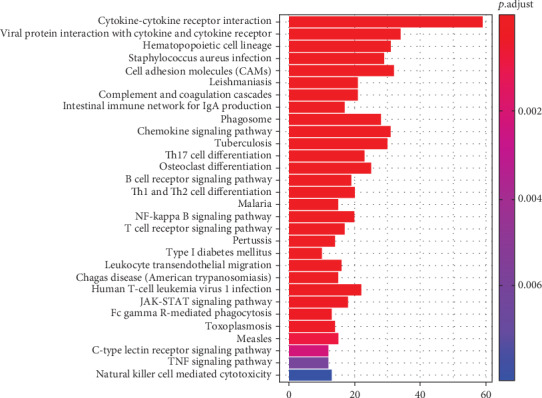
KEGG pathway enrichment analysis of DEGs obtained from TCGA.

KEGG pathway analysis revealed that 30 pathways were significantly enriched. Regarding the top five most enriched pathways, 59 DEGs were enriched in cytokine–cytokine receptor interaction pathways and 34 DEGs were enriched in viral protein interaction with cytokine and cytokine receptor pathways. In addition, 31 DEGs were enriched each in hematopoietic cell lineage and chemokine signaling pathways, whereas 30 DEGs were enriched in tuberculosis-related pathways (Figure 4). These results illustrate that all the pathways derived from the KEGG analysis are associated with immune responses.

### 3.5. Correlation between Individual DEG Expression and Overall Survival

**Figure 5:**
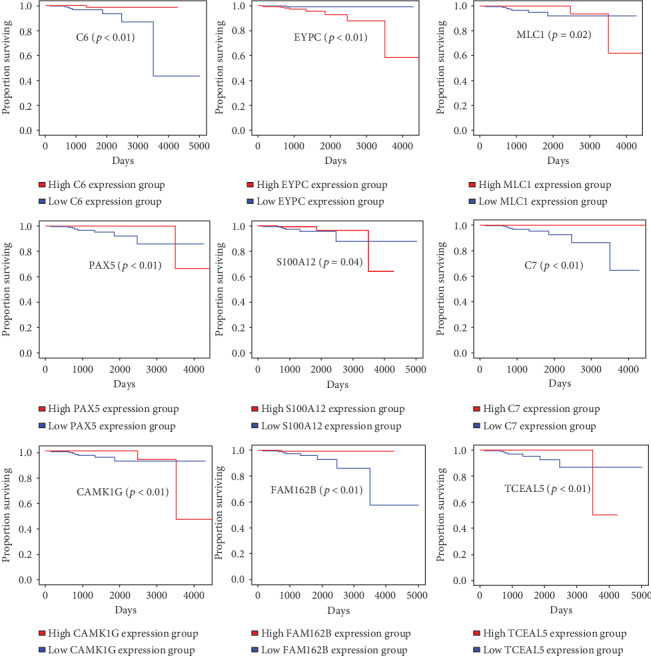
Correlations between DEG expression and overall survival. The survival curves of selected DEGs from the high (red line) and low (blue line) gene expression groups were generated using the weighted Kaplan–Meier analysis method (*p* < 0.05 in the log-rank test).

To explore the potential relationship between individual DEGs and overall survival, weighted Kaplan–Meier survival curves were generated from the TCGA data. The weighted Kaplan–Meier survival analysis alone with multivariate analysis identified five immune score-associated DEGs which were significantly associated with overall survival of PRAD patients. Four DEGs, *C6*, *S100A12*, *MLC1*, and *PAX5*, were positive prognostic factors, and *EPYC* was a negative prognostic factor for overall survival (*p* < 0.05 for all cases, weighted log-rank test, Supplementary Table 3, Figure 5). We also identified that seven stromal score-associated DEGs were significantly associated with overall survival. Of them, *EPYC*, *MLC1*, and *PAX5* were the common DEGs related to overall survival. The other four DEGs, *C7*, *FAM162B*, *CAMK1G*, and *TCEAL5*, were significantly predictive of favorable overall survival (*p* < 0.05 for all cases, weighted log-rank test, Supplementary Table 4, Figure 5).

### 3.6. PPI Network Analysis among DEGs of Prognostic Value

**Figure 6:**
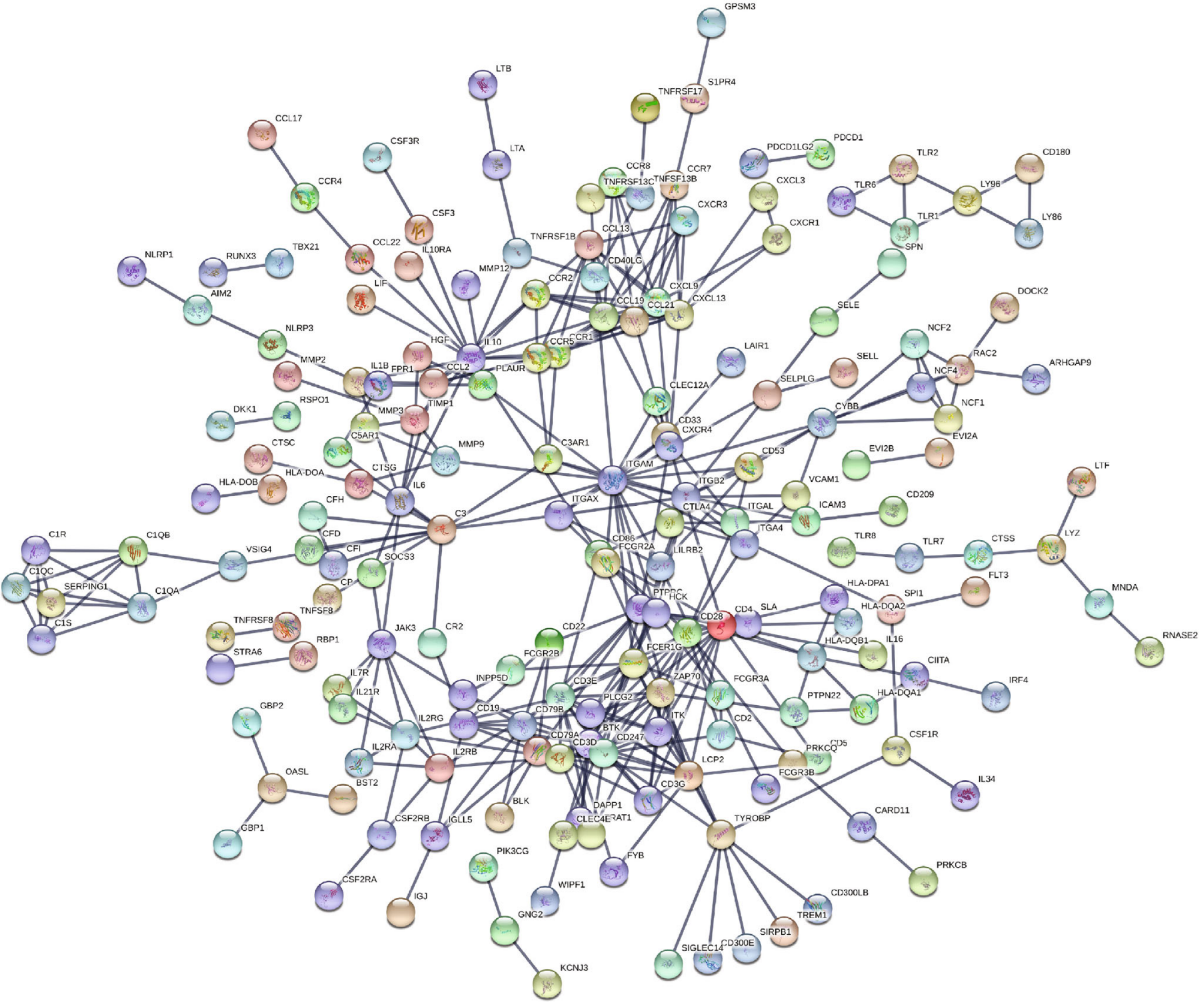
PPI network analysis of the 524 intersected genes between the immune and stromal score groups.

**Figure 7:**
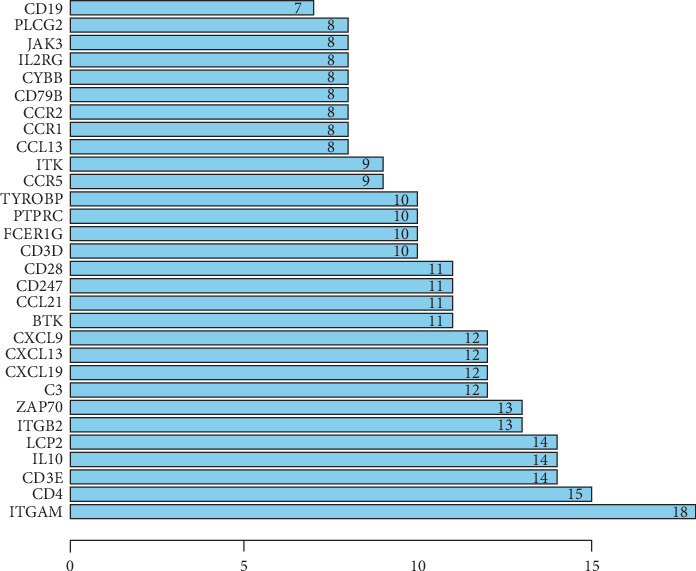
The 30 hub genes in the PPI network.

To better explore and understand the interactions among the identified DEGs, the PPI network of DEGs was constructed using the STRING tool. The network comprised 186 nodes and 364 edges. The top 30 DEGs with high degrees of connectivity were selected for analysis (Figure 6). The hub genes were ITGAM, CD4, CD3E, IL-10, LCP2, ITGB2, ZAP-70, C3, CCL19, CXCL13, CXCL9, BTK, CCL21, CD247, CD28, CD3D, FCER1G, PTPRC, TYROBP, CCR5, ITK, CCL13, CCR1, CCR2, CD79B, CYBB, IL2RG, JAK3, PLCG2, and CD19 (Figure 7). These prominent nodes had the most associations with other genes, indicating that they might play crucial roles in the prognosis of PRAD.

## 4. Discussion

The present study is aimed at identifying genes, which are related to the tumor microenvironment and closely related to the overall survival rate of PRAD. First, we analyzed the relationship between the T clinical stage of PRAD and immune scores (or stromal scores), discovering that the T clinical stage of PRAD is closely related to the tumor microenvironment. The sample size for T4 is far low than other T stages in our study; more comprehensive analysis and further exploration with large sample sizes are needed in the future studies. Immune and stromal cells have been proposed to be valuable for tumor diagnosis and prognosis evaluation. As indicated by the GO enrichment analysis, the function of immune cells and ECM is involved in the construction of the tumor microenvironment in patients with PRAD. Furthermore, the enriched KEGG pathways of the DEGs included cytokine–cytokine receptor interaction, viral protein interaction with cytokine and cytokine receptor, hematopoietic cell lineage, and chemokine signaling. KEGG pathways of infectious diseases such as malaria, toxoplasmosis, and Chagas disease were also correlated with DEGs in PRAD. It has been known that viral infection such as human papillomavirus increases the risk of PRAD [23]. The viral infection-related regulatory cytokines (IL-1beta) or tumor necrosis factor and many genes in Th1 and Th2 differentiation pathways were upregulated in the high immune score group and played an important role in host-pathogen interaction.

Next, we constructed PPI modules, all of which were related to immune/inflammatory responses. ITGAM, CD4, CD3E, IL-10, LCP2, ITGB2, ZAP-70, C3, CCL19, and CXCL13 were the top 10 hub genes in the PPI analysis, suggesting that these genes have a large number of interactions with other genes. Therefore, these genes may act as key genes in the PPI network. Moreover, these genes may play important roles in promoting tumor angiogenesis (CXCL13, ZAP-70, and CCL19), tissue remodeling (ITGAM and ITGB2), and immunosuppression (CD4 and IL-10) in cancer cell lines or samples [24–30]. In recent years, an increasing number of studies demonstrate that these inflammatory cytokines and anti-inflammatory cytokines play a key role in malignant tumors [31, 32] and the crosstalk between the cancer cells and the tumor stroma mainly comprising the basement membrane, fibroblasts, extracellular matrix, immune cells, and vasculature, to large extent, contributes to the progression of tumors and their metastasis [33, 34]. In particular, ITGAM and CXCR4 have attracted our attention. As illustrated in the PPI network, CXCR4 as a chemokine receptor has been found to be upregulated in cancer metastasis, and it has been used as a prognostic marker in various types of cancer, including leukemia, breast cancer, and prostate cancer [35–37]. As a major surface antigen family on human leukocyte family member, previous studies have reported the key role of ITGAM in the development and prognosis of human leukemia [38], ovarian cancer [39], and colorectal cancer [40]. ITGAM has been found to be upregulated PRAD after analyzing the patient samples [41], but there was no research on the role of ITGAM gene in PRAD, indicating the need for additional research to clarify its role in the prognosis of PRAD.

Research on gene expression and the overall survival rates of patients with PRAD has been conducted on a large scale, obtaining breakthrough results. We successfully extracted 9 genes involved in protein and immune responses by analyzing patients according to high and low immune scores. Patients with PRAD who carried these genes had significantly predictive overall survival, suggesting they may become potential prognostic biomarkers in PRAD. Many of these experiments were performed using in vitro tumor cell lines, animal tumor models, and a small number of patient tumor samples. However, PRAD and its microenvironment are extremely complex, requiring more comprehensive analyses and further exploration, including studies with larger sample sizes. Fortunately, the rapid development of TCGA provides us with a platform and foundation for further analysis.

The interaction between PRAD and its tumor microenvironment has serious effects on tumor evolution, further affecting tumor resistance, recurrence, and overall prognosis. Wang et al. [42] have provided a detailed description of the mechanism by which the activation of tumor-inherent genes affects the tumor microenvironment. The present study focused on the genetic characteristics of the tumor microenvironment, which affect the development and prognosis of PRAD. Our results may also provide a foundation for further studies on the tumor microenvironment of PRAD.

## 5. Conclusions

In summary, we used TCGA for functional enrichment analysis. Using the relationships of the immune score, based on the ESTIMATE method, with the T clinical stage and prognosis of PRAD, we extracted a list of genes related to the microenvironment of PRAD. These genes may be helpful for explaining the prognosis of PRAD. Some previously neglected genes may emerge as biomarkers for PRAD. Finally, further studies of these genes can provide a more comprehensive understanding of the potential relationship between the prognosis of PRAD and the tumor microenvironment.

## Data Availability

The data used to support the findings of this study are available from the corresponding author upon request.

## Abbreviations

PRAD: Prostate adenocarcinoma
TCGA: The Cancer Genome Atlas
GO: Gene Ontology
PPI: Protein-protein interaction
KEGG: Kyoto Encyclopedia of Genes and Genomes
STRING: Search Tool for the Retrieval of Interacting Genes/Proteins.

## Supplementary Materials

Supplementary Materials Supplementary Table 1: clinical data of prostate adenocarcinoma (PRAD) obtained from The Cancer Genome Atlas. Supplementary Table 2: linear regression model and correlation analyses between immune/stromal scores, overall survival, and potential confounders. Supplementary Table 3: survival analyses between patients' overall survival and DEG expression levels associated with immune scores. Supplementary Table 4: survival analyses between patients' overall survival and DEG expression levels associated with stromal scores.
