# Supplementary material for: Database Mining of Genes of Prognostic Value for the Prostate Adenocarcinoma Microenvironment Using the Cancer Gene Atlas
Source: Biomed Res Int. 2020 May 18;2020:5019793. doi: 10.1155/2020/5019793 (PMC7251429; doi:10.1155/2020/5019793)
Supplement: Supplementary Materials — Supplementary Table 1: clinical data of prostate adenocarcinoma (PRAD) obtained from The Cancer Genome Atlas. Supplementary Table 2: linear regression model and correlation analyses between immune/stromal scores, overall survival, and potential confounders. Supplementary Table 3: survival analyses between patients' overall survival and DEG expression levels associated with immune scores. Supplementary Table 4: survival analyses between patients' overall survival and DEG expression levels associated with stromal scores. [file 5019793.f1.zip › 5019793.f1.pdf]

Supplementary Table1. Clinical data of prostate adenocarcinoma (PRAD) obtained from The Cancer Genome Atlas

| Characteristic | Total |
|---|---|
| Cohort size[a], n | 490 |
| Age, years | 41–78 |
| T stage, n (%) | |
| pT2 | 187 (38.16%) |
| pT3 | 286(58.37%) |
| pT4 | 10 (2%) |
| Unknown | 7 (1.42%) |
| N stage, n (%) | |
| N0 | 340 (69.39%) |
| N1 | 78 (15.92%) |
| Unknown | 72 (14.69%) |

[a]Clinical data of three patients with PRAD were not available.
