# Supplementary material for: Database Mining of Genes of Prognostic Value for the Prostate Adenocarcinoma Microenvironment Using the Cancer Gene Atlas
Source: Biomed Res Int. 2020 May 18;2020:5019793. doi: 10.1155/2020/5019793 (PMC7251429; doi:10.1155/2020/5019793)
Supplement: Supplementary Materials — Supplementary Table 1: clinical data of prostate adenocarcinoma (PRAD) obtained from The Cancer Genome Atlas. Supplementary Table 2: linear regression model and correlation analyses between immune/stromal scores, overall survival, and potential confounders. Supplementary Table 3: survival analyses between patients' overall survival and DEG expression levels associated with immune scores. Supplementary Table 4: survival analyses between patients' overall survival and DEG expression levels associated with stromal scores. [file 5019793.f1.zip › 5019793.f2.pdf]

Supplementary Table2. Linear regression model and correlation analyses between immune/stromal scores, overall survival and potential confounders.

	StromalScore				ImmuneScore			
	T value	P value	Correlation coefficient	P value	T value	P value	Correlation coefficient	P value
T clinical stage	2.92	<0.01	0.18	<0.01	2.26	0.02	0.14	<0.01
Age	1.52	0.13			1.89	0.06		
Sample size	0.41	0.69			-0.51	0.61		
