# Supplementary material for: Database Mining of Genes of Prognostic Value for the Prostate Adenocarcinoma Microenvironment Using the Cancer Gene Atlas
Source: Biomed Res Int. 2020 May 18;2020:5019793. doi: 10.1155/2020/5019793 (PMC7251429; doi:10.1155/2020/5019793)
Supplement: Supplementary Materials — Supplementary Table 1: clinical data of prostate adenocarcinoma (PRAD) obtained from The Cancer Genome Atlas. Supplementary Table 2: linear regression model and correlation analyses between immune/stromal scores, overall survival, and potential confounders. Supplementary Table 3: survival analyses between patients' overall survival and DEG expression levels associated with immune scores. Supplementary Table 4: survival analyses between patients' overall survival and DEG expression levels associated with stromal scores. [file 5019793.f1.zip › 5019793.f3.pdf]

Supplementary Table3. survival analyses between patients' overall survival and DEG expression levels associated with immune scores

| | Kaplan-Meier survival analysis | | | Multivariate survival analysis | | | |
|---|---|---|---|---|---|---|---|
| | Cutoff | Chisq | P value | OR | 2.5%CI | 97.5%CI | P value |
| C6 | 0.04 | 8.08 | <0.01 | 0.13 | 0.02 | 1.00 | 0.04 |
| S100A12 | 0.10 | 4.42 | 0.04 | 0.24 | 0.06 | 1.00 | 0.04 |
| MLC1 | 0.14 | 5.78 | 0.02 | 0.25 | 0.08 | 0.84 | 0.02 |
| EPYC | 0.02 | 8.76 | <0.01 | 492423642.31 | 240285146.81 | 1009138711.77 | <0.01 |
| PAX5 | 0.11 | 8.97 | <0.01 | 0.12 | 0.02 | 0.70 | 0.02 |

Note: OR, odds ratio, CI, confidence interval
