# Supplementary material for: Database Mining of Genes of Prognostic Value for the Prostate Adenocarcinoma Microenvironment Using the Cancer Gene Atlas
Source: Biomed Res Int. 2020 May 18;2020:5019793. doi: 10.1155/2020/5019793 (PMC7251429; doi:10.1155/2020/5019793)
Supplement: Supplementary Materials — Supplementary Table 1: clinical data of prostate adenocarcinoma (PRAD) obtained from The Cancer Genome Atlas. Supplementary Table 2: linear regression model and correlation analyses between immune/stromal scores, overall survival, and potential confounders. Supplementary Table 3: survival analyses between patients' overall survival and DEG expression levels associated with immune scores. Supplementary Table 4: survival analyses between patients' overall survival and DEG expression levels associated with stromal scores. [file 5019793.f1.zip › 5019793.f4.pdf]

Supplementary Table4. survival analyses between patients' overall survival and DEG expression levels associated with stromal scores

	Kaplan-Meier survival analysis			Multivariate survival analysis			
	Cutoff	Chisq	P value	OR	2.5%CI	97.5%CI	P value
C7	10.07	18.53	<0.01	<0.01	<0.01	<0.01	<0.01
MLC1	0.14	8.49	<0.01	0.25	0.08	0.84	0.02
EPYC	0.02	6.71	0.01	492423634.00	240285131.15	1009138743.46	<0.01
FAM162B	1.04	12.09	<0.01	0.12	0.02	0.93	0.04
CAMK1G	0.42	9.07	<0.01	0.25	0.07	0.84	0.02
PAX5	0.11	7.37	0.01	0.12	0.02	0.70	0.02
TCEAL5	0.49	12.20	<0.01	0.12	0.02	0.64	0.01
